# Causal associations between biomarkers and depression: A Mendelian randomization study

**DOI:** 10.1097/MD.0000000000045105

**Published:** 2025-10-10

**Authors:** Jian Guo, Yang Jiang, Kaiqin Chen, Jingwen Li, Xuefeng Wang

**Affiliations:** a Department of Neurosurgery, The Fourth Affiliated Hospital of Harbin Medical University, Harbin, Heilongjiang Province, China; b Department of Neurosurgery, Xiang’an Hospital of Xiamen University, Xia Men, Fu Jian Province, China; c Department of Nursing, Xiang’an Hospital of Xiamen University, School of Medicine, Xiamen University, Xiamen, China; d Department of Neurosurgery, The First Hospital of Qiqihar, Qiqihar, Heilongjiang Province, China.

**Keywords:** biomarkers, depression, Mendelian randomization

## Abstract

Depression is one of the most prevalent and debilitating mental health conditions worldwide. Biomarkers have received considerable attention in depression research, to provide breakthroughs in early diagnosis, pathophysiological understanding, and personalized treatment. We employed Mendelian randomization (MR) methods to examine the causal connection between biomarkers and genetic predisposition to depression. Leveraging a substantial cohort from publicly accessible genome-wide association study datasets of European populations, we analyzed MR, utilizing the inverse variance weighting model as the primary approach. Additionally, we evaluated heterogeneity and horizontal pleiotropy. Our MR analysis revealed significant associations between biomarkers and depression. The inverse variance weighting model indicated that urinary potassium (odds ratio [OR] = 0.670, *P* = .012, β = −0.4), peak expiratory flow (OR = 0.886, *P* = .011, β = −0.143), cholesterol levels (OR = 0.935, *P* = .00508, β = −0.0674), and direct low-density lipoprotein levels (OR = 0.925, *P* = .00303, β = −0.0782) were associated with a reduced risk of depression. Conversely, triglyceride levels (OR = 1.05, *P* = .0246, β = 0.0489) were associated with a higher risk of developing depression. This study found that urinary potassium, peak expiratory flow, cholesterol levels, and direct low-density lipoprotein levels were associated with a reduced risk of depression. In contrast, triglyceride levels were correlated with a heightened risk of depression.

## 1. Introduction

Depression is one of the most prevalent and debilitating mental health conditions worldwide. Severe cases are characterized by profound sadness and a loss of interest, often accompanied by feelings of guilt, hopelessness, lack of energy and concentration, sleep disturbances, changes in appetite, and suicidal ideation.^[1]^ Epidemiological studies show that the prevalence of depression varies significantly across different regions and is closely linked to socioeconomic status, life events, and genetic factors.^[2]^ In recent years, the incidence of depression has steadily risen, particularly due to increasing social stress, lifestyle changes, and the global impact of the COVID-19 pandemic.^[3]^

In recent years, biomarkers have received considerable attention in depression research, to provide breakthroughs in early diagnosis, pathophysiological understanding, and personalized treatment. Key biomarkers in depression include neurotransmitters, inflammatory markers, neurotrophic factors, and neuroimaging features. Studies have shown that inflammatory responses are often elevated in individuals with depression, with significantly increased levels of serum C-reactive protein and pro-inflammatory cytokines, including interleukin-6 and tumor necrosis factor-α. Moreover, neurotrophic factors like brain-derived neurotrophic factor are often reduced, possibly linked to impaired neuroplasticity and dysfunction.^[4]^ Neuroimaging research indicates that depression is linked to structural and functional impairments in areas such as the prefrontal cortex, hippocampus, and amygdala.^[5]^ These biomarkers support early diagnosis and offer the potential for personalized treatment and outcome prediction. Nevertheless, the reliance on observational studies in this field often poses significant challenges, primarily attributable to confounding influences and the possibility of reverse causality.

In this context, Mendelian randomization (MR) provides distinct methodological advantages. MR utilizes genetic variations as instrumental variables (IVs), providing a powerful method to clarify causal associations between exposures and outcomes while reducing confounding factors and biases commonly found in observational studies. In this research, the MR framework is employed to investigate the potential causal links between 44 biomarkers and depression.

## 2. Methods

### 2.1. Research methods

We employed the MR approach to investigate the potential causal links between 44 biomarkers and depression. The MR framework leverages genetic variants as proxy markers for potential risk factors. Three core assumptions must be satisfied to validate the reliability of causal conclusions drawn from IVs. Genetic variants must be directly associated with the exposure of interest. Genetic variants must not be associated with confounding variables that could influence the exposure-outcome relationship. The association between the exposure and the outcome must be mediated solely through the genetic variants, without alternative pathways.

### 2.2. Data sources

Summary statistics for depression were sourced from a genome-wide association study (GWAS) database. The dataset included 59,333 cases and 434,831 controls, all of European ancestry. Depression was defined according to endpoint criteria established by the clinical expert group of the FinnGen study, consisting of renowned specialists in relevant medical disciplines.^[6]^ The FinnGen study has established a comprehensive framework for defining medical conditions, thereby ensuring the consistency and reliability of diagnostic criteria.^[7,8]^ Data for the 44 biomarkers were derived from the seminal work conducted by Joelle Mbatchou et al.^[9]^

### 2.3. Selection of instrumental variables (IVs)

In MR analysis, IVs are used as mediators between exposure and outcome to explore their causal relationship. IVs are typically genetic variations, with single-nucleotide polymorphisms (SNPs) being the most commonly used. In line with previous studies,^[10]^ we set the significance threshold for selecting IVs associated with each trait at *P* < 5 × 10⁻^8^. To account for linkage disequilibrium among SNPs, we applied an *r*² threshold with a significance threshold of 0.001 and a clumping distance set at 10,000 kb. Before each analysis, SNPs associated with the outcome (*P* < 5 × 10⁸) were excluded.

### 2.4. Statistical methods

Statistical analyses were conducted using R software. The primary analyses were performed with the TwoSampleMR package,^[11]^ employing inverse variance weighting (IVW)^[12]^ and weighted median^[13]^ to investigate the causal associations between 44 biomarkers and depression. Cochran *Q* statistic was applied to assess the variability or inconsistency among the IVs. The IVW method is widely regarded as the primary approach for inferring causality due to its high sensitivity in detecting causal effects. The MR-Egger approach accounted for possible horizontal pleiotropy, where the intercept was used to measure pleiotropy.^[14]^ Additionally, the Mendelian Randomization Pleiotropy RESidual Sum and Outlier method refined the analysis by identifying and excluding outliers attributable to pleiotropy. The statistical significance of the results was defined as *P* < .05.

## 3. Results

We investigated the effects of 44 biomarkers on depression. The SNPs used ranged from 12 to 439, with individuals of European ancestry in the exposures numbering between 332,739 and 406,552. The outcome dataset comprised 59,333 depression cases and 434,831 controls of European ancestry from the FinnGen biobank, with minimal overlap between exposure and outcome populations (Table 1).

**Table 1:** Information on exposure and outcome datasets (GAWS ID).

	Exposure/outcome/GAWS ID	Sample size (European)
Exposure	White blood cell leukocyte count (UKB data field 30,000) ‖ id:ebi-a-GCST90013976	396,621
	Red blood cell erythrocyte count (UKB data field 30010) ‖ id:ebi-a-GCST90013977	396,625
	Haemoglobin concentration (UKB data field 30020) ‖ id:ebi-a-GCST90013978	396,624
	Mean corpuscular volume (UKB data field 30040) ‖ id:ebi-a-GCST90013979	396,624
	Platelet count (UKB data field 30080) ‖ id:ebi-a-GCST90013980	396,621
	Mean platelet thrombocyte volume (UKB data field 30100) ‖ id:ebi-a-GCST90013981	396,616
	Lymphocyte count (UKB data field 30120) ‖ id:ebi-a-GCST90013982	395,949
	Monocyte count (UKB data field 30130) ‖id: ebi-a-GCST90013983	395,949
	Neutrophil count (UKB data field 30140) ‖ id:ebi-a-GCST90013984	395,949
	Eosinophil count (UKB data field 30150) ‖ id:ebi-a-GCST90013985	395,949
	Basophil count ‖ id:ebi-a-GCST90013986	395,949
	Creatinine enzymatic in urine (UKB data field 30510) ‖ id:ebi-a-GCST90013987	396,837
	Potassium in urine (UKB data field 30520) ‖ id:ebi-a-GCST90013988	395,995
	Sodium in urine (UKB data field 30530) ‖ id:ebi-a-GCST90013989	396,020
	Albumin levels (UKB data field 30600) ‖ id:ebi-a-GCST90013990	357,968
	Alkaline phosphatase levels (UKB data field 30610) ‖ id:ebi-a-GCST90013991	389,883
	Alanine aminotransferase levels (UKB data field 30620) ‖ id:ebi-a-GCST90013992	389,733
	Apolipoprotein A levels (UKB data field 30630) ‖ id:ebi-a-GCST90013993	355,859
	Apolipoprotein B levels (UKB data field 30640) ‖ id:ebi-a-GCST90013994	388,022
	Peak expiratory flow (UKB data field 3064) ‖ id:ebi-a-GCST90013995	374,599
	Aspartate aminotransferase levels (UKB data field 30650) ‖ id:ebi-a-GCST90013996	388,490
	Direct bilirubin levels (UKB data field 30660) ‖ id:ebi-a-GCST90013997	332,739
	Urea levels (UKB data field 30670) ‖ id:ebi-a-GCST90013998	389,608
	Calcium levels (UKB data field 30680) ‖ id:ebi-a-GCST90013999	357,831
	Cholesterol levels (UKB data field 30690) ‖ id:ebi-a-GCST90014000	389,864
	Creatinine levels (UKB data field 30700) ‖ id:ebi-a-GCST90014001	389,678
	C reactive protein levels (UKB data field 30710) ‖ id:ebi-a-GCST90014002	389,057
	Cystatin C levels (UKB data field 30720) ‖ id:ebi-a-GCST90014003	389,834
	Gamma glutamyltransferase levels (UKB data field 30730) ‖ id:ebi-a-GCST90014004	389,672
	Glucose levels (UKB data field 30740) ‖ id:ebi-a-GCST90014005	357,580
	Glycated hemoglobin HbA1c levels (UKB data field 30750) ‖ id:ebi-a-GCST90014006	389,889
	High-density lipoprotein cholesterol levels (UKB data field 30760) ‖ id:ebi-a-GCST90014007	357,810
	IGF 1 (UKB data field 30770) ‖ id:ebi-a-GCST90014008	387,834
	Direct low-density lipoprotein levels (UKB data field 30780) ‖ id:ebi-a-GCST90014009	389,189
	Phosphate levels (UKB data field 30810) ‖ id:ebi-a-GCST90014010	357,302
	Sex hormone binding globulin levels (UKB data field 30830) ‖ id:ebi-a-GCST90014011	354,620
	Total bilirubin levels (UKB data field 30840) ‖ id:ebi-a-GCST90014012	388,303
	Testosterone levels (UKB data field 30850) ‖ id:ebi-a-GCST90014013	353,805
	Triglyceride levels (UKB data field 30870) ‖ id:ebi-a-GCST90014014	389,562
	Urate levels (UKB data field 30880) ‖ id:ebi-a-GCST90014015	389,404
	Vitamin D levels (UKB data field 30890) ‖ id:ebi-a-GCST90014016	373,045
	Diastolic blood pressure automated reading (UKB data field 4079) ‖ id:ebi-a-GCST90014017	385,801
	Systolic blood pressure automated reading (UKB data field 4080) ‖ id:ebi-a-GCST90014018	385,798
	Hand grip strength left (UKB data field 46) ‖ id:ebi-a-GCST90014019	406,552
Outcome	Depression (Finngen Round 12 Release)	59,333 cases of European ancestry and 434,831 controls of European ancestry

GWAS = genome-wide association studies, IEU = integrative epidemiology unit, NA = not applicable; More information about exposure, mediating factors,and outcomes is available at the IEU OpenGWAS project (https://gwas.mrcieu.ac.uk/) and the Finngen (https://risteys.finngen.fi/). Data on depression can be downloaded from this link: https://storage.googleapis.com/finngen-public-data-r12/summary_stats/release/finngen_R12_F5_DEPRESSIO.gz

A 2-sample MR causal analysis was performed using IVW, weighted median, and MR-Egger methods. Table S1, Supplemental Digital Content, https://links.lww.com/MD/Q275 summarizes the results, including β values and odds ratios (ORs). After excluding outliers identified by the Mendelian Randomization Pleiotropy RESidual Sum and Outlier method, the number of SNPs ranged from 11 to 434 (Table S2, Supplemental Digital Content, https://links.lww.com/MD/Q275).

Following outlier exclusion, we refined biomarker selection based on IVW method *P*-values and excluded biomarkers exhibiting horizontal pleiotropy. This process identified 5 biomarkers associated with depression (Table 2). Specifically, potassium in urine (OR = 0.670, *P* = .012, β = −0.4), peak expiratory flow (OR = 0.886, *P* = .011, β = −0.143), cholesterol levels (OR = 0.935, *P* = .00508, β = −0.0674), and direct low-density lipoprotein levels (OR = 0.925, *P* = .00303, β = −0.0782) were associated with a reduced risk of depression. Conversely, triglyceride levels (OR = 1.05, *P* = .0246, β = 0.0489) were associated with a higher likelihood of developing depression.

**Table 2:** Biomarkers associated with depression were identified by excluding outliers detected through the MR-PRESSO method, refining the selection based on *P*-values from the IVW method, and omitting biomarkers with evidence of horizontal pleiotropy.

Serial Number	Outcome	exposure	Number of SNPs	IVW_ β	IVW_se	IVW_OR	IVW_lci95	IVW_uci95	IVW_pval	IVW_q_value	egger_intercept	se	*P*
1	Depression ‖ id:F5_DEPRESSIO	Potassium in urine (UKB data field 30520) ‖ id:ebi-a-GCST90013988	11	−4.00E−01	1.59E−01	6.70E−01	4.90E−01	9.16E−01	1.20E−02	1.32E−01	−2.10E−02	1.68E−02	2.43E−01
2	Depression ‖ id:F5_DEPRESSIO	Peak expiratory flow (UKB data field 3064) ‖ id:ebi-a-GCST90013995	119	−1.43E−01	5.68E−02	8.66E−01	7.75E−01	9.68E−01	1.15E−02	1.32E−01	−3.33E−03	2.80E−03	2.38E−01
3	Depression ‖ id:F5_DEPRESSIO	Cholesterol levels (UKB data field 30690) ‖ id:ebi-a-GCST90014000	171	−6.74E−02	2.40E−02	9.35E−01	8.92E−01	9.80E−01	5.08E−03	1.12E−01	−1.65E−03	1.27E−03	1.96E−01
4	Depression ‖ id:F5_DEPRESSIO	Direct low density lipoprotein levels (UKB data field 30780) ‖ id:ebi-a-GCST90014009	152	−7.82E−02	2.64E−02	9.25E−01	8.78E−01	9.74E−01	3.03E−03	1.12E−01	−8.12E−04	1.30E−03	5.32E−01
5	Depression ‖ id:F5_DEPRESSIO	Triglyceride levels (UKB data field 30870) ‖ id:ebi-a-GCST90014014	242	4.89E−02	2.17E−02	1.05E + 00	1.01E + 00	1.10E + 00	2.46E−02	2.16E−01	−3.47E−04	9.77E−04	7.23E−01

We identified 5 biomarkers associated with depression by first excluding outliers detected through the MR-PRESSO method, then refining the selection based on *P*-values from the inverse variance weighted (IVW) method, and finally eliminating biomarkers that exhibited horizontal pleiotropy. The IVW method, recognized for its high sensitivity in detecting causal relationships, was the primary approach used to determine causality. To account for horizontal pleiotropy, the MR-Egger method was employed, as it accommodates non-zero intercepts. Horizontal pleiotropy is indicated when the “egger_intercept,” “se,” and “pval” columns report a *P*-value below .05.

IVW = inverse variance weighted method; MR_Egger = MR-Egger method; SNPs = single-nucleotide polymorphisms; OR = odds ratio; lci95 = lower 95% confidence interval; uci95 = upper 95% confidence interval; q_value = adjusted *P*-value using the Benjamini–Hochberg false discovery rate method.

No associations were observed for the remaining 39 biomarkers, including markers related to blood cell counts (e.g., leukocyte count, erythrocyte count, platelet count), enzymatic activity (e.g., alkaline phosphatase, alanine aminotransferase), and electrolyte levels (e.g., sodium and creatinine in urine). For the full list of null findings, see Table S2, Supplemental Digital Content, https://links.lww.com/MD/Q275.

## 4. Discussion

The MR analysis identified significant associations between urinary potassium, peak expiratory flow, cholesterol levels, direct low-density lipoprotein (LDL) levels, triglyceride levels, and depression. While these findings are noteworthy, the potential causal relationships between other biomarkers, such as blood cell counts and enzymatic activity, and depression cannot be definitively excluded. MR analyses were repeated whenever outliers were detected to address the influence of outliers, with the updated results from the IVW model serving as the primary basis for evaluating causal relationships.

Previous studies have indicated a strong relationship between nutrition, inflammatory status, and depression.^[15]^ A meta-analysis examining the relationship between metabolic syndrome and the incidence of major depressive disorder (MDD) in elderly and non-elderly populations from Europe, the United States, Australia, and Japan reported a 49% increase in the incidence of MDD among individuals with metabolic syndrome.^[16]^ Metabolic syndrome includes a range of conditions such as insulin resistance, central obesity, impaired glucose tolerance, elevated triglyceride levels, low high-density lipoprotein (HDL) cholesterol, nonalcoholic fatty liver disease, and hypertension. Depression, a complex psychiatric disorder, has been increasingly linked to metabolic dysfunction. Evidence suggests a negative correlation between LDL cholesterol and total cholesterol levels with depression, whereas elevated triglyceride levels appear to be associated with an increased risk of depression. LDL cholesterol, a critical component of lipid metabolism, is often referred to as “bad cholesterol” due to its role in atherosclerosis.^[17]^ Emerging evidence suggests that LDL cholesterol may exert broader effects, including associations with mood disorders such as depression. Some studies have further explored these associations. For example, Xiaoyi Qi et al illustrated a direct, linear dose–response association between the ratio of non-HDL cholesterol to HDL cholesterol and the risk of depression.^[18]^ However, findings on non-HDL cholesterol and depression remain inconsistent. A cohort study observed higher LDL-C/HDL-C ratios in patients with major depressive episodes compared to healthy individuals,^[19]^ whereas a meta-analysis reported lower serum LDL-C levels in individuals with depression.^[20]^ A recent cross-sectional study using data from the Korea national health and nutrition examination survey further highlighted that while total cholesterol/HDL-C and LDL-C/HDL-C ratios were not significantly associated with depression, the triglyceride/HDL-C ratio demonstrated a significant positive association.^[21]^ Pro-inflammatory states associated with depressive episodes may arise from reduced HDL cholesterol and elevated LDL cholesterol levels. These changes can contribute to endothelial damage and the subsequent release of pro-inflammatory mediators such as interleukin-6, C-reactive protein, and soluble endothelial adhesion molecules. Additionally, activated immune cells release IL-1β, which stimulates the production of interferon-γ and tumor necrosis factor-α, both of which are typically elevated in patients with MDD.^[22]^

Our study identified a potential association between triglyceride levels and depression. Charilaos Chourpiliadis et al^[23]^ reported that elevated triglyceride levels were associated with a 15% increased risk of depression, anxiety, and stress-related disorders. These results indicate that increased triglyceride levels may play a role in the onset of depression and other associated psychiatric disorders. Similarly, a cohort study from the Netherlands^[24]^ demonstrated that a higher triglyceride-to-HDL ratio was linked to an 89% increased risk of major depressive disorder. This relationship may stem from metabolic disturbances caused by insulin resistance, which has been shown to promote neuroinflammation, a key feature in the neurobiological framework of depression.^[25,26]^ The inflammation hypothesis of depression further posits that pro-inflammatory cytokines can reduce serotonin levels, impair neurogenesis, and disrupt synaptic plasticity, pathophysiological changes closely associated with depression.^[27–29]^ Additionally, insulin resistance has been implicated in the dysregulation of the hypothalamic-pituitary-adrenal axis, particularly through glucocorticoid imbalances, which are recognized contributors to depressive pathology.^[26,30]^

Our study further identified an association between peak expiratory flow and a reduced risk of depression. Peak expiratory flow is closely linked to pulmonary function, with higher values indicating better lung health. A review involving 1161,632 participants highlighted the protective effects of maintaining moderate to high levels of pulmonary health against depression.^[31]^ Impaired lung function can lead to cerebral hypoxia and metabolic dysregulation,^[32]^ which may trigger a pro-inflammatory state characterized by the release of numerous pro-inflammatory cytokines. This heightened systemic inflammatory response has been associated with the onset of depressive symptoms.^[33]^ Additionally, a cross-sectional study based on data from the national health and nutrition examination survey revealed a significant negative correlation between forced vital capacity and depression.^[34]^

Current research is divided on the association between gender and depression. Although some investigative studies have pointed out that gender differences may affect the incidence and severity of depression (such as a higher reporting rate among women), a cross-sectional study of Indonesian students has shown no significant association between gender and depression levels.^[35,36]^ This contradiction was further confirmed in Jacob German study, the start time of treatment for patients with depression did not vary by gender.It is worth noting that factors such as the characteristic differences formed between men and women during the process of gender socialization (such as emotional expression patterns), cognitive attitudes towards mental health (such as stigma), and depressive attribution patterns (such as internal and external attribution tendencies) may lead to gender differences in self-reported data by influencing the behavior of seeking help rather than the nature of the disease.^[37,38]^ Therefore, when interpreting the gender differences in the incidence of depression, response biases (such as the low willingness to report caused by social norms in men and the greater tendency of women to label emotions) should be incorporated into the analytical framework rather than simply attributed to biological differences.

Our study has several limitations. First, due to the lack of summary-level GWAS data stratified by sex, we were unable to conduct sex-specific analyses. Second, depression is classified into mild, moderate, and severe forms, but in the absence of such data, we could not assess the effects of biomarkers on different severities of depression. Furthermore, although the MR approach effectively reduces confounding and reverse causality, it still relies on strong genetic IVs. Consequently, future research should incorporate larger sample sizes and a broader range of genetic variations to enhance the reliability and generalizability of our findings.

## 5. Conclusion

This study found that urinary potassium, peak expiratory flow, cholesterol levels, and direct LDL levels were associated with a reduced risk of depression. In contrast, elevated triglyceride levels were associated with a higher risk of depression. These results provide valuable insights into the metabolic pathways contributing to depression and could serve as a basis for developing future therapeutic approaches. Further studies are needed to confirm the involvement of these biomarkers in depressive episodes and to explore their potential for therapeutic use.

## Acknowledgments

Thank you to all the medical staff who contributed to the maintenance of the medical record database (FAERS, UKB, EBI, and IEU).

## Author contributions

**Conceptualization:** Jian Guo.

**Data curation:** Jian Guo, Yang Jiang, Kaiqin Chen, Jingwen Li, Xuefeng Wang.

**Formal analysis:** Jian Guo, Yang Jiang, Kaiqin Chen, Jingwen Li, Xuefeng Wang.

**Funding acquisition:** Jian Guo, Jingwen Li.

**Validation:** Jian Guo.

**Writing – original draft:** Jian Guo, Kaiqin Chen, Jingwen Li, Xuefeng Wang.

**Writing—review & editing:** Jian Guo, Yang Jiang, Jingwen Li, Xuefeng Wang.

## Supplementary Material


